# Poly(ADP-ribose) polymerase 1 in genome-wide expression control in *Drosophila*

**DOI:** 10.1038/s41598-020-78116-5

**Published:** 2020-12-03

**Authors:** Guillaume Bordet, Niraj Lodhi, Danping Guo, Andrew Kossenkov, Alexei V. Tulin

**Affiliations:** 1 Fox Chase Cancer Center, Philadelphia, PA USA; 2 Department of Biomedical Sciences, School of Medicine and Health Sciences, University of North Dakota, 501 North Columbia Road, Stop 9061, Grand Forks, ND 58202 USA; 3 The Wistar Institute, Philadelphia, PA USA

**Keywords:** Genetics, Functional genomics, Gene expression

## Abstract

Poly(ADP-ribose) polymerase 1 (PARP-1) is a nuclear enzyme involved in DNA repair and transcription regulation, among other processes. Malignant transformations, tumor progression, the onset of some neuropathies and other disorders have been linked to misregulation of PARP-1 activity. Despite intensive studies during the last few decades, the role of PARP-1 in transcription regulation is still not well understood. In this study, a transcriptomic analysis in *Drosophila melanogaster* third instar larvae was carried out. A total of 602 genes were identified, showing large-scale changes in their expression levels in the absence of PARP-1 in vivo. Among these genes, several functional gene groups were present, including transcription factors and cytochrome family members. The transcription levels of genes from the same functional group were affected by the absence of PARP-1 in a similar manner. In the absence of PARP-1, all misregulated genes coding for transcription factors were downregulated, whereas all genes coding for members of the cytochrome P450 family were upregulated. The cytochrome P450 proteins contain heme as a cofactor and are involved in oxidoreduction. Significant changes were also observed in the expression of several mobile elements in the absence of PARP-1, suggesting that PARP-1 may be involved in regulating the expression of mobile elements.

## Introduction

The poly(ADP-ribose) polymerase (PARP) enzyme family has been extensively studied during the last two decades. Initially recognized for their role in DNA repair^1^, PARPs have been shown to be involved in many other biological processes, including chromatin structure regulation, as well as transcriptional and translational activation and repression^2,3^. Several studies have revealed that members of the PARP family play key roles in the initiation and progression of malignant tumors, showing the clinical relevance of PARPs and leading to the development of PARP inhibitors for cancer treatment^4–6^. Despite intensive research, the mechanism underlying the ability of PARP to regulate chromatin structure and transcription remains poorly understood.

The PARP family is responsible for the poly ADP-ribosylation (PARylation) of its target proteins, a type of post-translational modification involving the polymerization of ADP-ribose units. This modification is highly negatively charged and can lead to a repulsion of the target proteins from the DNA^7^. The PARP family includes 17 different members in mammals and only 2 members in *Drosophila*; each member presents a specific expression pattern and subcellular localization^8,9^. The most studied member of this family is PARP-1. This 116 kDa nuclear protein is responsible for the majority of poly(ADP-ribose) (PAR) syntheses in both mammals and *Drosophila*^8^. In *Drosophila*, only PARP-1 is localized in the nucleus, while PAPR-5 is located in the cytoplasm^10^. PARP-1 possesses three major domains: a DNA binding domain, an auto-modification domain, and a catalytic domain with low basal activity^11^. Its auto-modification domain acts as a negative feedback loop. When active, PARP-1 modifies itself, which results in its own repulsion from DNA. The three domains allow PARP-1 to modulate a wide range of processes involving genomic DNA. For example, activation of PARP-1 catalytic activity has been shown to promote chromatin loosening, both in mammals and *Drosophila*^9,12^, suggesting an involvement of PARP-1 in gene expression activation. The removal of PARP-1 from chromatin can result in chromatin loosening as well, suggesting that PARP-1 can also be involved in maintaining gene repression^13^. Although the expression of several genes has been shown to be affected by PARP-1 knockdown in Human^2^, PARP-1 in *Drosophila melanogaster* has only been confirmed to regulate the expression of *Heat shock protein 70* (*Hsp70*) during heat shock in vivo^10,14^ and to regulate innate immunity genes expression during microbial infection^15^.

To identify other genes regulated by PARP-1, we compared the in vivo expression profile of *Drosophila melanogaster* flies between wild type and mutant fly strain based on a complete *parp-1* knockout. We focused on *Drosophila* for two reasons. First, only one nuclear PARP is found in *Drosophila*, which allows us to avoid redundancy. Second, flies with complete *parp-1* knockout are viable up to third instar larvae/ pupation stage^16^, which allows us to perform in vivo tests in *parp-1* knockout condition. For this study, we followed a whole organism approach to detect large-scale organismal changes in the expression profiles of all genes. This approach does not detect low-scale, tissue-specific changes, particularly when the vectors of change differs among different tissues. We found that several genes were misregulated in the absence of PARP-1 relative to wild type. Using Gene Set Enrichment analysis (GSEA) software, we identified the functional groups of these misregulated genes. We observed that genes downregulated in the absence of PARP-1 generally belonged to functional groups other than those of the upregulated genes.

## Results

### Comparison of the expression profile of larvae in the presence and absence of PARP-1

**Figure 1:**
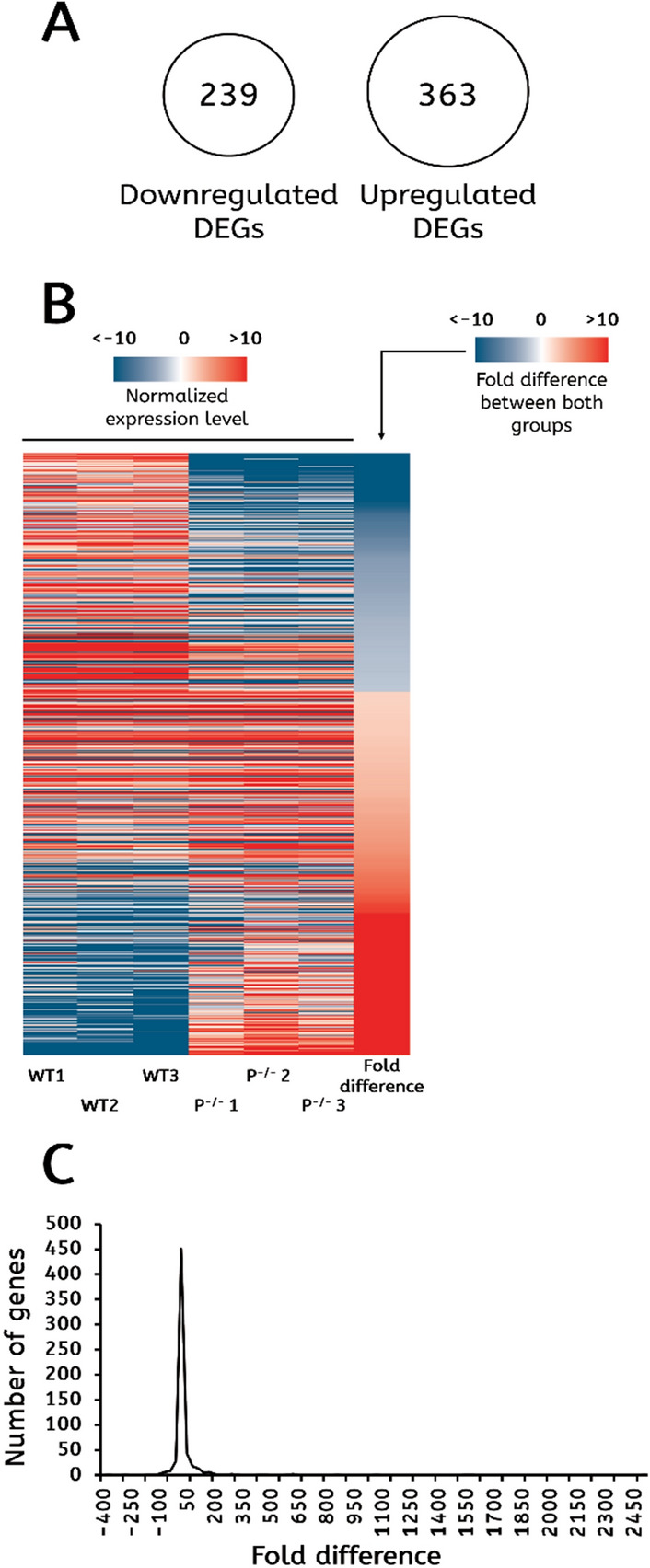
An overview of the differentially expressed genes (DEGs) between the wild-type and *parp-1*^−/−^ groups: (**A**) Comparison between the number of DEGs downregulated in *parp-1*^−/−^ larvae (downregulated DEGs) and the number of DEGs upregulated in *parp-1*^−/−^ larvae (upregulated DEGs). (**B**) Heat map representing the differences in expression level for all DEGs between wild type (WT) and the *parp-1*^−/−^ (P^−/−^) flies. Biological replicates are shown separately. The expression level of each gene was normalized to the average level of expression of all genes. Negative values (in blue shading) represent the downregulated DEGs, while positive values (in red shading) represent the upregulated DEGs. The last column represents the fold difference between the wild type and *parp-1*^−/−^ groups. The fold difference was measured as the negative expression level in the wild type larvae divided by the expression level in the *parp-1*^−/−^ larvae when the expression was higher in the wild type group (shown in blue) and the expression level in *parp-1*^−/−^ larvae divided by the expression level in wild type larvae when the expression was higher in the *parp-1*^−/−^ group (shown in red). (**C**) Distribution of the 602 DEGs, depending on the fold difference of their expression level between the wild type and *parp-1*^−/−^ groups.

To compare the expression profile in the presence and absence of PARP-1, we used the *parp-1*^*C03256*^ fly strain, which is a complete *parp-1* knockout. Flies with this mutation can survive up to third instar larvae/late pupation stage without exhibiting any developmental delay compared to *yellow white* larvae^16^. We collected a mixture of 150 male and female third instar larvae for the *parp-1*^*C03256*^ and *yellow white* strains, hereinafter noted as the *parp-1*^−/−^ and wild type groups, respectively. We then split these 150 larvae into three biological replicates. The expression profile was determined using a microarray chip and was realized in three biological replicates to control for random fluctuations. The fluorescence intensity for each one of 18,453 probes is presented in our raw data as a log_2_ value (Supplementary Table 1). The fold difference was calculated for each gene based on fluorescence level in each biological replicate before any normalization (Fig. 1). Differentially expressed genes (DEGs) were identified based on the following criteria: (1) the fold difference had to be higher than two, (2) a two-tailed *t*-test based on the change of expression between the wild-type and *parp-1*^*−/−*^ groups relative to the standard deviation of all measurements had to show a *p* value lower than 0.05, and (3) the False Discovery Rate (FDR) had to be lower than 15% (see “Materials and Methods” for details).

Our analysis identified 602 genes that were differentially expressed between wild- type and *parp-1*^−/−^ larvae (Supplementary Table 2). Among them, 239 were downregulated in *parp-1*^−/−^ larvae compared to wild type. We refer to these genes as “downregulated DEGs”. The remaining 363 genes were upregulated in* parp-1*^−/−^ larvae compared to wild type, and we refer to these genes as “upregulated DEGs” (Fig. 1A). The three biological replicates produced similar results for each gene, suggesting that the expression level was stable in our two separate groups (Fig. 1B). The range of differential expression level was extremely broad between the wild-type and *parp-1*^−/−^ groups, ranging from negative 387-fold for *cg7900*, which has no known function, to 2505-fold for the mobile element *springer*. However, 451 out of our 602 DEGs (75%) had a fold difference ranging between − 10 and 20 only (Fig. 1C). To test for reproducibility, we selected 10 genes that were significantly misregulated in our microarray and tested them by qRT-PCR. All 10 DEGs we selected presented similar results between microarray and qRT-PCR, suggesting that our results are, indeed, reproducible (Supplementary Table 3). To determine which cellular functions were more affected by the absence of PARP-1, we next carried out a functional analysis of DEGs.

### Cellular functions of DEGs

**Figure 2:**
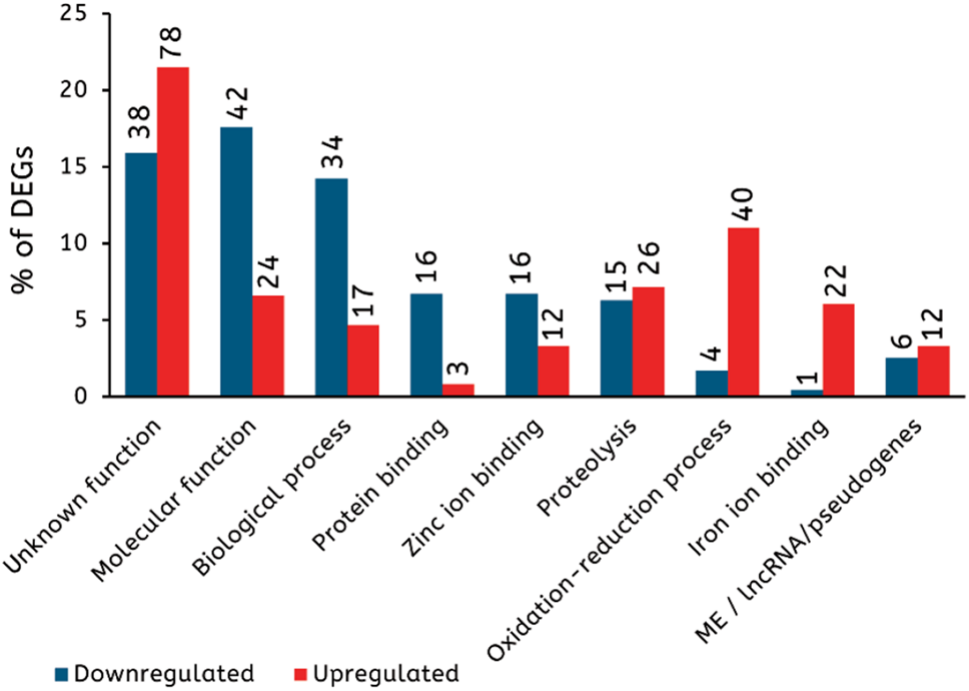
An overview of the main cellular functions of the 602 genes differentially expressed between the wild type and *parp-1*^−/−^ groups. Only categories that include at least 5% of the downregulated or upregulated DEGs are shown. The Y axis corresponds to the percentage of genes belonging to each functional category among the downregulated (blue) or the upregulated (red) DEGs. The black number at the top of each bar corresponds to the number of DEGs belonging to each specific process. *ME* mobile element. *lncRNA* long non-coding RNA.

To identify the function of DEGs, we extracted gene data on cellular functions from Flybase^17^ and compared them to our list of DEGs. Among the 602 DEGs, 486 possess at least one known cellular function, whereas the cellular function of the remaining 116 was unknown. Among DEGs with at least one known cellular function, 201 were downregulated and 285 were upregulated. Among DEGs with unknown function, 38 were downregulated and 78 upregulated (Supplementary Table 4). The 486 DEGs with a known cellular function belonged to eight functional categories (Fig. 2). Interestingly, only one category was represented in the same proportion among the downregulated and upregulated DEGs. DEGs involved in ‘Proteolysis’ represent 6.2% (15/239) of the downregulated DEGs and 7.2% (26/363) of the upregulated DEGs. The other seven categories were not represented in a similar proportion between the downregulated and upregulated DEGs. Four of these categories were better represented among the downregulated DEGs. The most represented is the category ‘Molecular function’, which comprised 17.6% (42/239) of the downregulated DEGs against 6.6% (24/363) of the upregulated DEGs. The second most represented category is ‘Biological process’ comprising 14.2% (34/239) of the downregulated DEGs against 4.7% (17/363) of the upregulated DEGs. Genes involved in ‘Protein binding’ represent 6.7% (16/239) of the downregulated DEGs against 0.8% (3/363) of the upregulated DEGs. Genes involved in ‘Zinc ion binding’ represent 6.7% (16/239) of the downregulated DEGs compared to 3.3% (12/363) for the upregulated DEGs. Two categories were better represented among the upregulated DEGs. First, ‘Oxidation–reduction process’ comprises 1.7% (4/239) of the downregulated DEGs against 11% (40/363) of the upregulated DEGs. Second, genes involved in ‘Iron ion binding’ represent 0.4% (1/239) of the downregulated DEGs against 6.1% (22/363) of upregulated DEGs. We also noticed the presence of pseudogenes, long non-coding RNA and mobile elements among DEGs, which, together, represent 2.5% (6/239) of the downregulated DEGs and 3.3% (12/363) of upregulated DEGs. Taken together, these data show that prevalent functional categories tend to differ between downregulated and upregulated DEGs.

### Distribution of DEG types between the wild type and *parp-1*^−/−^ groups

**Figure 3:**
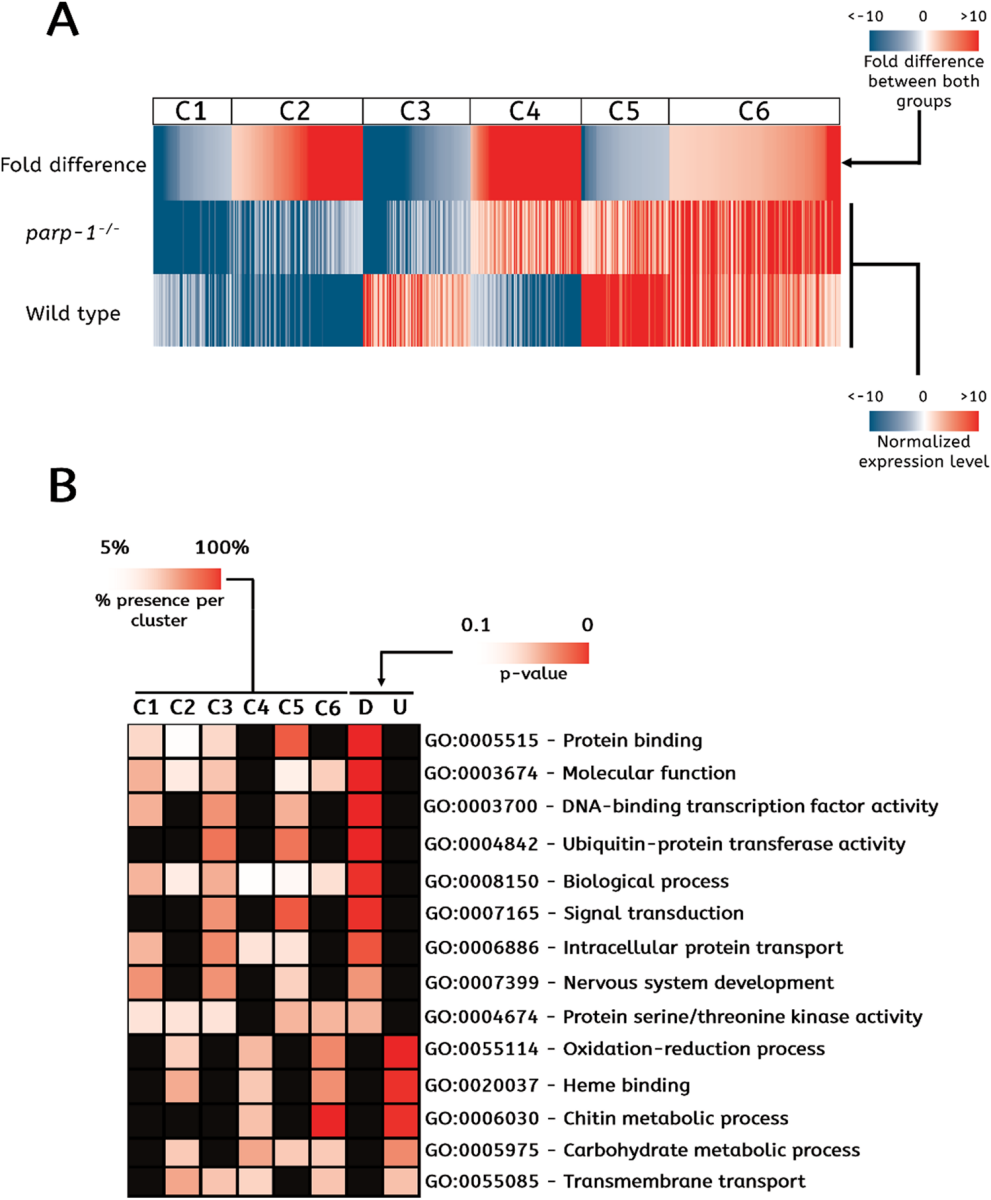
The functional classification of differentially expressed genes (DEGs) between the wild type and *parp-1*^−/−^ groups. (**A**) Heatmap of DEGs. Expression values for the wild type and *parp-1*^−/−^ groups are shown as normalized to the average expression level of all DEGs. Blue shading indicates that the expression level of a gene was lower than the mean, whereas red shading indicates a value higher than the mean expression level. The fold difference column corresponds to the ratio of the expression level of a gene between the wild type and *parp-1*^−/−^ groups. The blue shading represents downregulated genes in the *parp-1*^−/−^ group, whereas the red shading represents upregulated genes in the *parp-1*^−/−^ group. The 602 DEGs are sorted into six different clusters. (**B**) Gene Ontology-term (GO-term) overrepresentation among the downregulated or upregulated DEGs and the prevalence of each GO-term in each cluster. The six first columns correspond to the six clusters represented in (**A**) (C1 to C6). The significance of the overrepresentation of each GO-term in the wild type and *parp-1*^−/−^ groups is indicated by the *p* value in the two last columns (D for the downregulated DEGs and U for the upregulated DEGs). The heatmap below each cluster corresponds to the percentage of genes belonging to each GO-term that are present in that cluster.

To determine the role of PARP-1 in gene expression regulation of DEGs, we first normalized the expression level of every DEG to the average expression level of all DEGs. The wild-type group was normalized by the average expression level of all DEGs in the wild-type group, and the *parp-1*^−/−^ group was normalized by the average expression level of all DEGs in *parp-1*^−/−^. We then divided all DEGs into six clusters based on the difference of their expression level between the wild type and *parp-1*^−/−^ groups (Fig. 3A, Table 1). This clustering sorts DEGs according to their expression profile in both groups. The first cluster (C1) consists of 68 genes that had a normalized expression lower than average in both groups and that were downregulated in the *parp-1*^−/−^ group. The second cluster (C2) includes 115 genes with a lower than average normalized expression in both groups and that were upregulated in the *parp-1*^−/−^ group. The third cluster (C3) comprised 94 genes that had a lower normalized expression than the average in the *parp-1*^−/−^ group, but higher than the average in the wild type group. The fourth cluster (C4) includes 97 genes that had a higher expression than the average in the *parp-1*^−/−^ group and lower than the average in the wild-type group. The fifth cluster (C5) includes 77 genes that had a higher expression than the average in both groups and were downregulated in the *parp-1*^−/−^ group. The sixth cluster (C6) comprises the remaining 151 genes that had a higher expression level than the average in both groups and were upregulated in the *parp-1*^−/−^ group.

**Table 1:** Recapitulation of the composition of the different DEG clusters used in this study.

Cluster	Number of genes included	Expression in the wild-type group compared to average	Expression in the PARP −/− group compared to average	Down- or Upregulated in absence of PARP?
C1	68	Lower	Lower	Downregulated
C2	115	Lower	Lower	Upregulated
C3	94	Higher	Lower	Downregulated
C4	97	Lower	Higher	Upregulated
C5	77	Higher	Higher	Downregulated
C6	151	Higher	Higher	Upregulated

Clusters 1 and 2 include the DEGs with lower expression compared to the other genes in both the wild type and *parp-1*^−/−^ groups. Clusters 3 and 4 include DEGs with lower expression compared to the other genes in one group and a higher expression in the other group. Clusters 5 and 6 include DEGs with higher expression compared to other genes in both wild type and *parp-1*^−/−^ groups.

Only 183 out of 602 genes (30.4%) had a lower than average normalized expression in both the wild type and *parp-1*^−/−^ groups (C1 and C2), whereas 419 out of 602 (69.6%) had a higher than average expression in at least one of our groups (C3-6). Because it is mathematically easier to attain a large fold difference when the initial level of expression is low, the fluctuation levels in expression of the DEGs belonging to C1 and C2 may not necessarily be biologically relevant. Changes in the expression levels of DEGs belonging to C3 or C4 in the presence or absence of PARP-1 are the most biologically relevant because they present higher expression than average in one of the groups and lower expression than average in the other group. Finally, genes in C5 and C6 are also relevant because they present higher expression than average in both groups, making it mathematically harder to attain a significant fold difference between them. Clustering DEGs allows us to distinguish between the more and less biologically relevant DEGs based on changes in expression levels between the two groups.

### Functional classification of DEGs

To classify DEGs according to their function and to confirm that the functions of the downregulated DEGs differ from those of the upregulated DEGs, we performed gene set enrichment analysis using GSEA software (see “Materials and methods” and Supplementary Fig. 1). We selected the most significant Gene Ontology (GO)-term that included at least 4 DEGs (Fig. 3B). Interestingly, we found little overlap between the gene ontology (GO)-terms overrepresented among the up- and downregulated DEGs. This means that only a small proportion (4.2%) of downregulated DEGs was involved in a GO-term overrepresented among upregulated DEGs and only a small fraction (8.5%) of the upregulated DEGs was involved in a GO-term overrepresented among downregulated DEGs (data not shown). We identified nine significant GO-terms that mainly include downregulated DEGs (Fig. 3B). DEGs involved in ‘GO:0005515 Protein binding’ were overrepresented among downregulated DEGs with 13 out of 17 DEGs (76.5%) belonging to C3 or C5. DEGs involved in ‘GO:0003674 Molecular function’ and ‘GO:0008150 Biological process’ were overrepresented among downregulated DEGs with 23 out of 67 DEGs (34.3%) with 20 out of 52 DEGs (38.5%) belonging to C3 and C5, respectively. All DEGs involved in ‘GO:0003700 DNA-binding transcription factor activity’ and ‘GO:0004842 Ubiquitin-protein transferase activity’ were downregulated in *parp-1*^−/−^ larvae with 7 out of 10 DEGs (70%) and 6 out of 6 DEGs (100%) belonging to C3 or C5, respectively. Similarly, DEGs involved in ‘GO:0007165 Signal transduction’ were all downregulated DEGs with 7 out of 9 DEGs (77.8%) and 4 out of 7 DEGs (57.1%) belonging to C3 and C5, respectively. DEGs involved in ‘GO:0007399 Nervous system development’ were all downregulated in *parp-1*^−/−^ larvae with 3 out of 5 DEGs (60%) belonging to C3 and C5. Finally, DEGs involved in ‘GO:0006886 Intracellular protein transport’ and ‘GO:0004674 Protein serine/threonine kinase activity’ were overrepresented among downregulated DEGs with 4 out of 7 DEGs (57.1%) and 3 out of 7 DEGs (42.9%) belonging to C3 and C5, respectively.

We also found five significant GO-terms that mainly included upregulated DEGs. DEGs involved in ‘GO:0055114 Oxidation–reduction process’ were overrepresented among upregulated DEGs with 31 out of 44 DEGs (70.4%) belonging to C4 and C6, respectively. DEGs involved in ‘GO:0020037 Heme binding’ and ‘GO:0005975 Carbohydrate metabolic process’ were overrepresented among upregulated DEGs with 14 out of 22 DEGs (63.6%) and 5 out of 9 DEGs (55.5%) belonging to C4 and C6, respectively. All DEGs involved in ‘GO:0006030 Chitin metabolic process’ were upregulated in *parp-1*^−/−^ larvae with 8 out of 8 DEGs (100%) belonging to C4 and C6. Finally, DEGs involved in ‘GO:0055085 Transmembrane transport’ were overrepresented among upregulated DEGs with 9 out of 21 DEGs (42.8%) belonging to C4 and C6.

Taken together, these results show that several processes are differentially regulated in the absence of PARP-1. For example, DEGs involved in the GO-term ‘DNA-binding transcription factor activity’ were all downregulated in the absence of PARP-1, whereas DEGs involved in the GO-term ‘Heme binding’ were all upregulated in absence of PARP-1.

### DEGs with transcription factor activity are downregulated in the absence of PARP-1

The twelve DEGs involved in the GO-term ‘DNA-binding transcription factor activity’ were the only transcription factors we found to be misregulated in the absence of PARP-1; all of them were downregulated in the *parp-1*^−/−^ group (Table 2). Nine of these transcription factors are involved in neuronal development or axonal growth. Among the twelve misregulated transcription factors, we found three C2H2 zinc finger transcription factors; two of them were involved in neuronal development or axonal growth. Specifically, the gene coding for Longitudinals lacking (Lola) (cluster 1) and the gene coding for Castor (Cas) (cluster 3) were both downregulated in the absence of PARP-1. Lola is a transcription repressor involved in axonal growth and guidance in the *Drosophila* central nervous system (CNS)^18^. Lola is also important for programmed cell death in ovaries^19^ and in cell fate in eyes by antagonizing Notch induction^20^. Castor is involved in the temporal patterning of *Drosophila* CNS^21^.

**Table 2:** List of DEGs that code for a transcription factor.

*D. melanogaster* gene name	Fold difference	Cluster	Protein group	Human orthologue
Lola	− 3.25	1	C2H2 zinc finger transcription factor	ZBTB20
Ac	− 2.92	1	bHLH transcription factor	ASCL1
CG31365	− 2.71	1	C2H2 zinc finger transcription factor	BCL6B
Eip78C	− 63.68	3	C4 zinc finger ligand-dependent transcription factor	NR1D2
Cas	− 10.79	3	C2H2 zinc finger transcription factor	CASZ1
SoxN	− 6.75	3	High mobility group box transcription factor	SOX2
E(spl)m7-HLH	− 4.75	3	bHLH transcription factor	HES2
Repo	− 4.15	3	Homeodomain transcription factor	ALX3
Cyc	− 3.68	3	bHLH transcription factor	ARNTL
NFAT	− 5.52	5	Rel homology domain transcription factor	NFAT5
Slbo	− 2.44	5	Basic Leucine zipper transcription factor	CEBPD
E(spl)mα-BFM	− 2.26	5	bHLH transcription factor	/
Nelf-E	− 5.2	3	Member of the NELF complex	NELFE
Chm	− 3.47	5	Histone acetyl-transferase	KAT7
Med7	− 2.44	5	Member of the Mediator complex	MED7
mod(mdg4)	− 2.14	5	Other DNA binding domain transcription factor	BACH1

A negative fold difference means that the gene had a higher level of expression in the wild type group compared to that in the *parp-1*^−/−^ group. The second part of the table represents DEGs that play a role in transcriptional processes, but are not transcription factors.

We also found four basic helix-loop-helix (bHLH) transcription factors involved in neuronal development. The gene coding for Achaete (Ac) (cluster 1), the genes coding for two members of the Enhancer of Split family, E(spl)m7-HLH (cluster 3) and E(spl)mα-BFM (cluster 5), and the gene coding for Cycle (Cyc) (cluster 3) were all downregulated in the absence of PARP-1. Ac plays a role in the formation of neural precursors^22^ by serving as an antagonist to Notch signaling^23^. E(spl)m7-HLH and E(spl)m α-BFM are also involved in Notch signaling. More specifically, E(spl)m α-BFM plays a role in Notch lateral inhibition^24^, and E(spl)m7-HLH acts within the Notch pathway to repress neural fate^25^, but it has also been reported to interact with Ac^26^. Cyc is a circadian clock protein, which mediates several processes, including the olfaction rhythms of the antennal neurons^27^ and the interconnection of feeding and sleeping behavior^28^.

Three other transcription factors are involved in neuronal development. All were downregulated in the absence of PARP-1, including the gene coding for the high mobility group box transcription factor Soxneuro (SoxN) (cluster 3), the gene coding for the homeodomain transcription factor Reversed polarity (Repo) (cluster 3), and the gene coding for the Nuclear factor of activated T-cells transcription factor (NFAT) (Cluster 5). SoxN plays a role in the specification of neural progenitors in CNS^29^. This transcription factor is also important in the regulation of Wg/Wnt signaling activity^30^. Repo has been reported to participate in the maintenance of glial fate in the *Drosophila* nervous system^31^. NFAT plays an important role in several processes, including neuronal development and plasticity^32^.

The three remaining transcription factors downregulated in the absence of PARP-1 have not been reported to play a direct role in neuronal development. These included the gene coding for the C4zinc finger ligand-dependent transcription factor Ecdysone-induced protein 78C (Eip78C) (cluster 3), the gene coding for the basic leucine zipper transcription factor Slow border cells (Slbo) (cluster 5), and *cg31365* coding for a C2H2 zinc finger transcription factor (cluster 1). Eip78C plays a role in regulating chromosome puffs^33^ and ovarian germline stem cells^34^. Eip78C has also been reported to physically interact with SoxN and Cyc^35^. Slbo has been reported to participate in cell migration during oogenesis^36^. The transcription factor for the protein coded by *cg31365* relies on predicted functionality based on its sequence similarity with BCL6B, a human transcriptional repressor, but has never been confirmed in vitro^17^. Human BCL6B is a tumor suppressor that inhibits hepatocellular carcinoma metastasis^37^.

We identified four additional genes involved in the transcriptional process. Although not classified as transcription factors, they were still misregulated in the *parp-1*^−/−^ group; all were downregulated. These included the gene coding for the Negative elongation factor E (Nelf-E) (cluster 3), the gene coding for the histone acetyltransferase Chameau (Chm) (cluster 5), the gene coding for the Mediator complex subunit 7 (MED7) (cluster 5), and the gene coding for Modifier of mdg4 (Mod(mdg4)) (cluster 5). Nelf-E is a member of the NELF complex involved in pausing RNA polymerase II at several promoters, including *hsp70* promoter^38^. Nelf-E is involved in the activation of several key developmental genes during embryogenesis^39^. Chm is known to act along with the polycomb group to maintain *hox* gene silencing^40^. Chm also serves as a cofactor in the JNK/AP-1-dependent transcription during metamorphosis^41^. MED7 is a member of the mediator complex, which serves as a hub for transcriptional signaling events^42^. Modifier of mdg4 has 31 alternative splice products reported to participate in a range of processes, including chromosome segregation and synapse formation^43,44^.

Taken together, these results suggest that PARP-1 promotes expression of multiple transcription factors, as well as several genes mediating transcription. The absence of PARP-1 suppresses expression of several transcription factors important in neuronal development and axonal growth.

### DEGs coding for cytochrome P450 are upregulated in the absence of PARP-1

Among DEGs, we identified 22 genes involved in the ‘GO:0020037 Heme binding’ GO-term (Fig. 3B). All but one were upregulated in the *parp-1*^−/−^ group compared to wild type. The expression of these upregulated genes in the absence of PARP-1 varied from 2.56 for Cyp9f3Ψ to 1575.97 for Cyp6a17. We determined that 18 out of these 22 DEGs (81.8%) belonged to the cytochrome P450 family, three were related to the cytochrome b5 family, and the last one coding for peroxidase Globin 1 (Glob1) (cluster 6) was unrelated to the cytochrome family^45^ (Table 3).

**Table 3:** List of DEGs that code for heme-binding proteins.

*D. melanogaster* gene name	Fold difference	Cluster	Protein group	Human orthologue
Cyp4s3	5.41	2	Other cytochrome p450	CYP4A11
Cyp6a8	6.46	2	Other cytochrome p450	CYP3A4
Cyp12a5	8.58	2	Other cytochrome p450	CYP27A1
Cyp9b1	20.32	2	Other cytochrome p450	CYP3A5
Cyp6a2	20.93	2	Other cytochrome p450	CYP3A4
Cyp4d8	7.25	4	Other cytochrome p450	CYP4B1
Cyp4e3	28.37	4	Other cytochrome p450	CYP4B1
Cyp12c1	37.81	4	Other cytochrome p450	CYP24A1
Cyp6w1	434.77	4	Other cytochrome p450	CYP3A4
Cyp6a17	1575.97	4	Other cytochrome p450	CYP3A5
Cyp4p2	− 3.92	5	Other cytochrome p450	CYP4F2
Cyp9f3Psi	2.56	6	Other cytochrome p450	/
Cyp6d4	2.81	6	Other cytochrome p450	CYP3A4
Cyp28a5	3.35	6	Other cytochrome p450	TBXAS1
Cyp6a13	3.67	6	Other cytochrome p450	CYP3A4
Cyp4p1	3.80	6	Other cytochrome p450	CYP4B1
Cyp9c1	4.29	6	Other cytochrome p450	CYP3A4
Cyp12e1	4.49	6	Other cytochrome p450	CYP27B1
CG5157	10.50	2	Cytochrome b5. heme binding site	CYB5B
fa2h	20.97	2	Cytochrome b5. heme binding site	FA2H
Cyt-b5-r	5.43	6	Cytochrome b5. heme binding site	FADS1
glob1	4.78	6	Other peroxidase	CYGB

21 out of 22 DEGs are related to the cytochrome family. A positive fold difference means that this gene had a lower level of expression in the wild type group compared to that in the *parp-1*^−/−^ group. The first part of the table includes DEGs directly related to the cytochrome P450 family. The second part of the table includes DEGs coding for a protein with a cytochrome b5 heme-binding site. The last part for the table includes DEGs involved in heme-binding, but are not related to cytochrome family.

Cytochrome P450 proteins contain heme as a cofactor and are involved in oxidoreduction. These proteins are important in the clearance of several compounds and in hormone synthesis and breakdown^46^. Among DEGs related to the cytochrome P450 family, 14 out of 18 (77.8%) have at least a predicted function in the breakdown of toxic chemicals. Based on their sequence, seven have been predicted to play a role in the metabolism of insect hormones and the breakdown of toxic chemicals, although this function has still not been confirmed in vivo^17,47^. These included Cyp4s3 (cluster 2), Cyp9b1 (cluster 2), Cyp6a17 (cluster 4), Cyp4d8 (cluster 4), Cyp6d4 (cluster 6), Cyp28a5 (cluster 6), and Cyp6a13 (cluster 6). Cyp6a17 has been reported to play an important role in temperature preference behavior^48^. Seven have been shown to be involved in breakdown of toxic chemicals. Six are involved in Dichlorodiphenyltrichloroethane (DDT) resistance, including Cyp6a8 (cluster 2)^49^, Cyp6a2 (cluster 2)^49^, Cyp6w1 (cluster 4)^50^, genes coding for Cyp4p1 (cluster 6) and Cyp4p2 (cluster 5) that are organized in a cluster on DNA^51^, and the gene coding for Cyp9c1 (cluster 6)^52^. Finally, the gene coding for Cyp4e3 (cluster 4) plays a role in permethrin insecticide tolerance^53^. The last four DEGs coding for a member of the cytochrome P450 family (Cyp12e1, Cyp12a5, Cyp12c1, and Cyp9f3Ψ) do not have any predicted functions aside from heme-binding.

Three DEGs involved in the GO-term ‘Heme binding’ had a cytochrome b5 heme-binding site protein signature; however, these DEGs are not directly linked to the cytochrome P450 family, and all of them were upregulated in the absence of PARP-1. Cytochrome b5 proteins are hemoproteins involved in electron transport^54^. These three genes include the gene coding for the Fatty acid 2-hydrolase (Fa2h) (cluster 2), the gene coding for the Cytochrome b5-related (Cyt-b5-r) (cluster 6), and *cg5157* (cluster 2). Fa2h plays an important role in larvae survivability when a correct amount of sterol is lacking^55^. Cyt-b5-r is predominantly expressed in muscles^56^. The gene *cg5157* is predicted to possess a cytochrome b5 heme-binding site, but its function is unknown^17^.

Taken together, these results suggest that PARP-1 inhibits the expression of several cytochrome-related proteins. Most of them contribute to resistance against toxic chemicals, such as DTT or permethrin, suggesting that PARP-1 may contribute to the organismal response to toxins.

### Mobile elements tend to be upregulated in the absence of PARP-1

We also found eight mobile elements (Table 4, first panel) among DEGs. Three were downregulated in the *parp-1*^−/−^ group, and five were upregulated, ranging from − 33.96 for the mobile element *ivk* to 2505.47 for the mobile element *springer*. Five of these mobile elements are non-LTR retrotransposons belonging to the *LINE*-like-element superfamily^57^. *ivk* and *I-element* belong to Clade *I*, whereas *TART-element*, *He T-A,* and *juan* belong to the Clade *jockey*^57^. The three remaining MEs are LTR retrotransposons belonging to the *gypsy* superfamily^57^. *diver1* belongs to the Clade *roo*, *transpac* belongs to the Clade *17.6*, and *springer* belongs to the Clade *gypsy*^57^. We also found ten MEs that were upregulated in the *parp-1*^−/−^ group, ranging in fold difference from 2.32 to 35.32 (Table 4, second panel). For four, the *p* values comparing the significance of change of expression between the wild type and *parp-1*^−/−^ groups relative to the standard deviation of all measurements were below 0.05, but the FDRs were above 15%. For the other six MEs, *p* values were greater than 0.05. Among those ten MEs, nine were LTR retrotransposons belonging to the *gypsy* superfamily, and the last one was a non-LTR retrotransposon belonging to the *LINE*-like-element superfamily^57^. These results suggest that several mobile elements are misregulated in the absence of PARP-1 and that the majority of them were upregulated in the *parp-1*^−/−^ group.

**Table 4:**
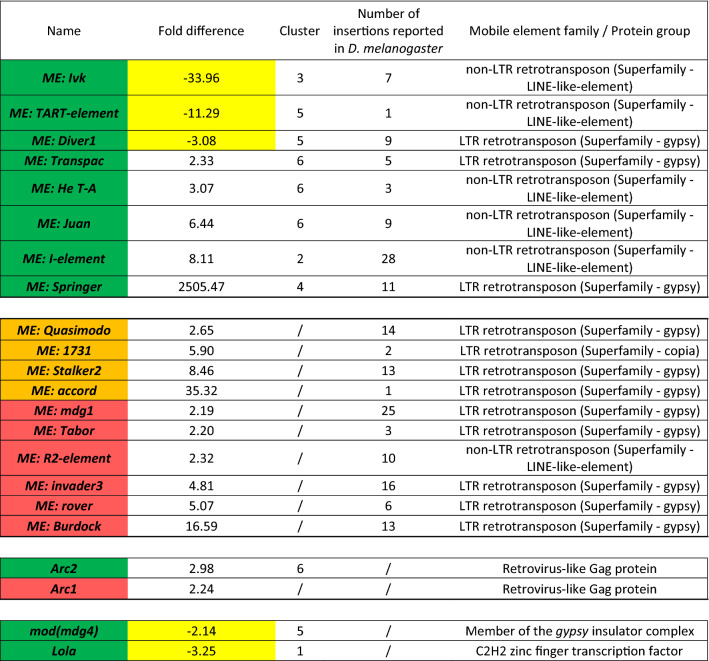
The first panel of the table lists mobile elements (MEs) misregulated in the absence of PARP-1.

The second panel represents MEs were misregulated in the absence of PARP-1, but presented an FDR > 15% or a *p* value > 0.05. The third panel lists genes that act like endogenous retroviruses. The fourth panel lists genes reported to play a role in the regulation of MEs. Green shading marks DEGs with a fold difference > 2.0, *p* values < 0.05, and FDR < 15%. Orange shading marks DEGs with fold difference > 2.0, *p* values < 0.05, and FDR > 15%. Red shading marks DEGs with fold difference > 2.0, *p* values > 0.05, and FDR > 15%. DEGs downregulated in the *parp-1*^−/−^ group are marked in yellow. The number of insertions reported in *Drosophila melanogaster* is based on *Kaminker *et al*.*^57^.

In addition, we found that the *arc2* gene was upregulated in the *parp-1*^−/−^ group compared to the wild-type group (fold difference of 2.98) (Table 4, third panel). Arc proteins are composed of Group-specific antigen (Gag)-like amino acid sequences typical of retroviruses and retrotransposons^58^. Interestingly, we also found that *arc1* was upregulated in the *parp-1*^−/−^ group. However, the difference was not statistically significant by having a *p* value greater than 0.05. PARP-1 might be important for repressing retrotransposon and retrotransposons-like gene expression.

Finally, we identified a number of genes involved in retrotransposon regulation among DEGs (Table 4, fourth panel). Among them is a gene coding for the transcription factor-like Modifier of mdg4 (Mod(mdg4)). Mod(mdg4) is a member of the *gypsy* insulator complex reported to repress the mobility of *P-element* transposon^59^. Consistent with the upregulation of most mobile elements, Mod(mdg4) was downregulated in the *parp-1*^−/−^ group. However, the other members of this complex (Su(hw), CP190, and Topors) were not misregulated in the absence of PARP-1^60^. Finally, the gene coding for the Lola transcription factor was downregulated in the absence of PARP-1. Lola has been reported to repress the expression of retrotransposons in CNS^61^. The downregulation of *lola* expression may be involved in the upregulation of retrotransposons in the absence of PARP-1. Taken together these findings suggest that mobile elements are mainly upregulated in the absence of PARP-1.

## Discussion

**Figure 4:**
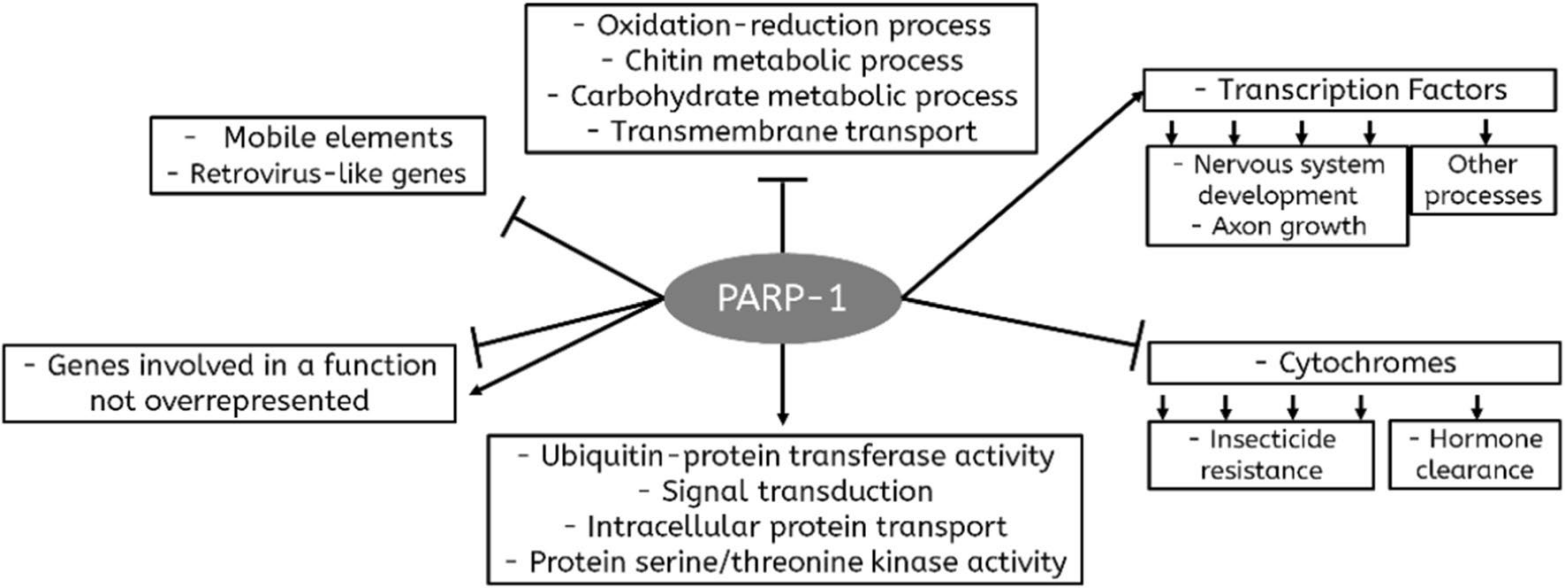
Diagram summarizing the role of PARP-1 in regulating processes in *Drosophila* at an organismal level according to this study. The sharp arrows represent groups of genes upregulated by PARP-1 activity, and the blunt arrows represent downregulated groups of genes.

Our results show that the absence of PARP-1 triggers large-scale changes in the expression of 602 genes in vivo, suggesting that PARP-1 mediates the expression profile of those genes. Among DEGs with known function, several gene-ontology terms were overrepresented, including ‘Signal transduction,’ ‘DNA-binding transcription factor activity,’ ‘Ubiquitin-protein transferase activity,’ ‘Nervous system development,’ ‘Oxidation–reduction process,’ ‘Heme binding,’ ‘Chitin metabolic process,’ and ‘Transmembrane transport’. We found little overlap between the gene-ontology terms overrepresented among the up- and downregulated DEGs. Therefore, it appears that PARP-1 stimulates the transcription of genes responsible for certain functions and inhibits the transcription of other functional gene groups. Thus, genes involved in ‘DNA-binding transcription factor activity,’ ‘Ubiquitin-protein transferase activity,’ ‘Signal transduction,’ ‘Nervous system development’ and ‘Intracellular protein transport’ were overrepresented among the downregulated DEGs, whereas genes involved in ‘Oxidation–reduction process,’ ‘Heme binding,’ ‘Chitin metabolic process,’ ‘Carbohydrate metabolic process’ and ‘Transmembrane transport’ were overrepresented among the upregulated DEGs (Fig. 4).

More than a half of DEGs with known functions were not involved in GO-terms overrepresented among DEGs, suggesting that many standalone genes are controlled by PARP-1, even though several major functional groups of genes with transcription profiles are regulated by PARP-1. Most genes regulated by PARP-1 do not appear to share their functions with one another. In those instances when a functional group of genes is regulated by PARP-1, all members of the group are regulated in a similar way (*i.e.,* up- or downregulated) (Fig. 4).

We also checked in the literature the phenotype of the knockdown/knockout of the ten most downregulated DEGs in *parp-1*^−/−^ group to see if they are compatible with the phenotype observed in *parp-1* mutant. Seven of them were knocked down using RNAi. For six of them, the flies are viable and do not exhibit any phenotype: CG7900 (− 387.5), alpha-Est2 (− 283.2), Obp57b (− 80.6)^62^, CG11893 (− 282.5), CG11034 (− 132.5) and CG44014 (− 90)^63^. The last one knocked down with RNAi is lethal before pupation (IntS12 (− 83.7))^63^, similar to *parp-1* knockout phenotype. Two of them were knocked out using gene trap and are lethal before pupation, Glut1 (− 88.5)^64^ and PGRP-LE (− 107.7)^65^. Finally, the flies knocked out for Lama (− 110) using enhancer trap are viable^66^. All these phenotype are compatible with *parp-1* knockout phenotype.

The transcription level of all twelve transcription factors found among DEGs was strongly downregulated in the absence of PARP-1. Eight of these transcription factors are directly involved in neuronal development and axonal growth. This finding is consistent with a study showing that post-mitotic neurons expressing RNAi against *parp-1* present defects in axonal outgrowth and branch patterning in vitro^67^. Taken together, these results suggest that PARP-1 stimulates the expression of transcription factors, which mediate neuronal development and axonal growth. However, we did not detect any misregulation in the expression of known downstream targets for these transcription factors in the absence of PARP-1. It is possible that our approach, which focuses on large-scale differences in the transcription profile, did not detect tissue-specific misregulation. Therefore, genes expressed in several tissues, but only misregulated in neurons or neuronal progenitors in the absence of functional PARP-1, did not show fold difference at transcription levels sufficient to be recognized as DEGs in our study. Another possibility is that the absence of misregulation of downstream targets in *parp-1*^−/−^ group may be due to the fact that many developmentally regulated transcription factors serve as a pioneer factors and downstream activation or repression requires the involvement of other factors that act later in the development^68^.

Aside from one DEG, all other DEGs shown to be related to the cytochrome P450 and Cytochrome-b5 families were upregulated in the absence of PARP-1. Among the 21 cytochrome-related DEGs, 14 have been reported as contributing to the clearance of toxic chemicals, including DTT and permethrin, suggesting that PARP-1 activity may reduce the resistance to toxic chemicals by limiting the expression of genes involved in the clearance of these toxic chemicals. Roles of most members of the cytochrome family listed in Table 3 remain poorly understood since seven only have predicted functions based on their DNA sequences. Apart from their role in toxic chemicals clearance, it is possible that all these genes are also involved in other processes, such as hormone synthesis/clearance.

Several mobile elements were also misregulated in the absence of PARP-1. Most were among the upregulated DEGs, consistent with one of our earlier studies that showed a de-repression of retrotransposons in the absence of PARP-1^69^. We also found that two known retrotransposon repressors were downregulated in the absence of PARP-1: Lola^61^ and Mod(mdg4)^59^. We then cannot exclude the possibility that the retrotransposon de-repression observed in *parp-1*^−/−^ group may be a consequence of the downregulation of the expression of retrotransposon repressors such as Lola and Mod(mdg4) rather than a direct upregulation of retrotransposon expression due to the absence of PARP-1.

### Possible mechanisms of action for PARP-1 in regulating expression

The main function of PARP-1 is the poly(ADP)ribosylation (PARylation) of target proteins. It is a post-translational modification involving the polymerization of ADP-ribose units. This modification is highly negatively charged and can lead to repulsion between the target proteins and DNA^7^. Two alternative mechanisms of PARP-1 involvement in the regulation of gene expression level are possible: (1) direct involvement through the regulation of chromosome condensation to facilitate or repress access to promoter of target genes or (2) indirect involvement through the PARylation of transcription factors/cofactors/insulators of the target genes. These two mechanisms are not mutually exclusive.

PARP-1 is involved in chromatin loosening through its activation by JIL-1^9^. Such PARP-1-mediated chromatin loosening could lead to activation of DEGs that were downregulated in the absence of PARP-1. Alternatively, because PARP-1 can compact chromatin in vitro^70^ and because we have demonstrated that PARP-1 is enriched during the interphase in regions where gene expression is silenced in vivo^71^, PARP-1 could potentially repress transcription by maintaining a compact chromatin state.

PARP-1 could also play an indirect role in regulating DEG expression through the PARylation of transcription factors/cofactors important for their correct expression^72^. It has also been reported that PARP-1 is involved in the PARylation of insulators that lead to their repulsion from DNA^73^. The insulator is then unable to block the enhancer/promoter interaction with its target gene, leading to the activation of the repressed gene.

## Materials and methods

### Drosophila melanogaster strains used

Flies were cultured on standard cornmeal-molasses-agar media at 22 °C, unless otherwise indicated. All the fly stocks listed below are isogenic. The fly stocks were generated by the standard genetic methods or obtained from the Bloomington Drosophila Stock Center and the Exelixis Collection at the Harvard Medical School. Genetic markers are described in Flybase^17^. The *parp-1*^*C03256*^ strain was generated in a single pBac-element mutagenesis screen^74^. Precise excision of *parp-1*^*C03256*^ was carried out using pBac transposase on *CyO* chromosome. Balancer chromosomes carrying *Kr::GFP*, i.e., *TM3*, *Sb*, *P{w* + *, Kr-GFP4}* and *FM7i*, *P{w1, Kr-GFP}*^75^, were used to identify heterozygous and homozygous *parp-1*^*C03256*^. The genetic background is similar in both *parp-1*^*C03256*^ and *yellow white* strain. Larvae from both genotypes were synchronized by placing adult flies in a fresh vial and letting them lay eggs for three hours before transferring them to another vial. 50 third instar larvae were collected for each biological replicate for both conditions without sex selection.

### RNA isolation and microarray

Total RNA was purified using the RNeasy Mini kit (Qiagen, Valencia, CA) after its isolation using TRIzol reagent (Life Technologies, Inc., Grand Island, NY). Microarray services, including quality control tests of the total RNA samples by Agilent Bioanalyzer and Nanodrop spectrophotometry, were provided by the UPENN Molecular Profiling Facility. All protocols followed the NuGEN Ovation User Guide and the Affymetrix GeneChip Expression Analysis Technical Manual. Briefly, 100 ng of total RNA were converted to first-strand cDNA using the reverse transcriptase primed by poly(T) and random oligomers that incorporated an RNA priming region. Second-strand cDNA synthesis was followed by ribo-SPIA linear amplification of each transcript using an isothermal reaction with RNase, RNA primer, and DNA polymerase (Ovation RNA Amplification System V2, NuGEN Inc., San Carlos CA, USA), and the resulting cDNA was assessed by Bioanalyzer, fragmented and biotinylated (Encore Biotin Module, NuGEN Inc., San Carlos CA); 3.75 μg of labeled cDNA were added to Affymetrix hybridization cocktails. Target hybridization was performed on GeneChip *Drosophila* Genome 2.0 arrays (Affymetrix Inc., Santa Clara CA, USA), according to the manufacturer’s procedures found in GeneChip Hybridization Oven 645, followed by washing and staining in GeneChip Fluidics Station 450. Data were acquired with the GeneChip Scanner 3000 7G (Affymetrix, Inc., Santa Clara, CA, USA). Three biological replicates per group were made (wild type and *parp-1* knockout).

### Quantitative RT-PCR assay

This assay was performed in triplicate. Twelve third instar larvae were collected for both wild type and *parp-1*^−/−^ groups. Total RNA was extracted from cells using the QIAshredder column and RNeasy kit (Qiagen). Contaminating genomic DNA was removed by the g-column provided in the kit. cDNA was obtained by reverse transcription using M-NLV reverse transcriptase (Invitrogen) Real-time PCR assays were run using SYBR Green master mix (Bio-Rad) and an Applied Biosystems StepOnePlus TM instrument. The amount of DNA was normalized using the difference in threshold cycle (*C*_*T*_) values (Δ*C*_*T*_) between *RpL32* and the different target genes.

The quantitative real-time PCR (qPCR) primer sequences for *Drosophila melanogaster* ribosomal protein L32 gene (*RpL32*) were 5′-GCTAAGCTGTCGCAACAAAT-3′ (forward) and 5′-GAACTTCTTGAATCCGGTGGG-3′ (reverse).

Sequences for *Cg3588* were 5′-CAAAGAACGGAGAACGGC-3′ (forward) and 5′-AATCCAAAGCCGCCAAAC-3′ (reverse).

Sequences for *Cyp6w1* were 5′-TTACATCTGGCAAGATCAAGC-3′ (forward) and 5′-TCACTTGGACTTCCGTACC-3′ (reverse).

Sequences for *NinaD* were 5′-GCCCCACATTTACCTTCATTG-3′ (forward) and 5′-AGAGATGTCCACCATTCGC-3′ (reverse).

Sequences for *alpha-Est7* were 5′-AACCTCGGCTTTGTGGAG-3′ (forward) and 5′-CTGAAGTAGGGCACATCGTAG-3′ (reverse).

Sequences for *Cg11893* were 5′- CAATGATGGTCTGTGGAAGC-3′ (forward) and 5′-CGTATTCGCTTTAACGGCC-3′ (reverse).

Sequences for *MtnC* were 5′-GCTGCGGAACAAACTGC-3′ (forward) and 5′-GCCATTCTTGCACACGC-3′ (reverse).

Sequences for *Cyc* were 5′-GCAAACGTCACCGATTGG-3′ (forward) and 5′-TCATCTTGTCCCGACGC-3′ (reverse).

Sequences for *Nfat* were 5′-AAAGACAGCCGGGTAAGGGAT-3′ (forward) and 5′-CAGGAACCATTTTGCCAGGAC-3′ (reverse).

Sequences for *Ac* were 5′-CAACGACGACGAGGAGTC-3′ (forward) and 5′-GCTGAAGCCATTGTTGACC-3′ (reverse).

Sequences for *Eip78C* were 5′-TCTACGATGTCATCATGTGCG-3′ (forward) and 5′-ACTGTGCTGGCAATCCCATTT-3′ (reverse).

### Microarray data analysis

Data analysis was performed using Partek Genomics Suite v6.5 to apply the GCRMA normalization algorithm. Differential expression analysis was conducted using Significance Analysis of Microarrays (SAM, v3.09) and 2-way ANOVA tool. SAM calculates a score for each gene based on the change in expression relative to the standard deviation of all measurements by computing a *t*-test based on the three biological replicates. SAM then performs a set of permutations to determine the false discovery rate (FDR) with an adjustment for multiple testing. The reported list of ranked genes has a ‘delta value’ which defines the threshold of false positive in the validated dataset, which was adjusted to a stringent FDR < 15%^76^. Affymetrix .cel files were imported into the Partek Genomic Suite (v6.5, Partek Inc., St. Louis, MO), and GCRMA normalization was applied. Data were exported to SAM (v3.09, Significance Analysis of Microarrays, Stanford University) to test for differential expression, yielding a fold change, a *p* value based on a *t*-test realized on the three biological replicates, and an FDR. The fluorescence intensity of each probe was recorded as a log_2_ value. To determine the expression level for every gene, the formula 2^X^ was used, where X is the average fluorescence intensity of the three biological replicates. The ratio between the expression level in the wild type and the *parp-1* knockout group, referred to as fold difference, was used to evaluate the changes of expression levels between the wild type and *parp-1* knockout groups for every gene. A negative value corresponds to a gene that is upregulated in wild-type larvae compared to *parp-1* knockout larvae, while a positive value corresponds to a gene upregulated in *parp-1* knockout larvae. 602 genes were found to be differentially expressed using cutoffs of twofold increase or decrease with a False Discovery Rate < 15% and a *p* value for the two-tailed *t*-test < 0.05. Using a combination of *p* value and FDR allows us to identify Differentially Expressed Genes (DEGs), while minimizing the number of false positives^76^. The fold difference establishes an arbitrary threshold for a difference of expression level between the wild type and *parp-1* knockout groups that we accepted as sufficient difference to consider a gene as misregulated between the two groups.

### DEGs cellular functions and GO-terms overrepresentation analysis

The main cellular functions of the genes differentially expressed between the wild- type and *parp-1* knockout groups was determined using PANTHER classification system^77,78^.

Overrepresentation of Gene Ontology-terms (GO-terms) among DEGs was determined using Gene Set Enrichment Analysis (GSEA) software, which has been described in *Subramanian *et al*.* and *Mootha *et al*.*^79,80^ (see Supplementary Fig. 1 for details). The metric used to rank genes has been set up on “Signal2Noise”. GO-terms with fewer than 4 DEGs were excluded during the analysis. Only the GO-terms with *p* value < 0.05 and FDR < 25% were considered as overrepresented in one of our groups. To avoid redundancy, when a GO-term was included in another GO-term, only the GO-term representing the smallest subset of genes was selected.

## Supplementary information

Supplementary Information.

Supplementary Table 1.

Supplementary Table 2.

Supplementary Table 3.

Supplementary Table 4.
