# Supplementary material for: Poly(ADP-ribose) polymerase 1 in genome-wide expression control in Drosophila
Source: Sci Rep. 2020 Dec 3;10:21151. doi: 10.1038/s41598-020-78116-5 (PMC7712786; doi:10.1038/s41598-020-78116-5)
Supplement: Supplementary file 1 — Supplementary Information. [file 41598_2020_78116_MOESM1_ESM.docx]

**Supplementary Appendix for**

**Poly(ADP-ribose) Polymerase 1 in genome-wide expression control in *Drosophila***

Guillaume Bordet^2^, Niraj Lodhi^1^, Danping Guo^2^, Andrew Kossenkov^3^, Alexei V. Tulin^2^

1 - Fox Chase Cancer Center, Philadelphia, PA

2 - University of North Dakota, Grand Forks, ND

3 - The Wistar Institute, Philadelphia, PA

**Corresponding author:** Alexei V. Tulin

Email: alexei.tulin@ndus.edu

**This PDF file includes:**

**Supplementary Tables 1 – 4 Legends**

**Supplementary Figure 1.** Examples of results obtained with Gene Set Enrichment Analysis (GSEA) software.

***Supplementary Table 1***

Microarray raw data. The first column corresponds to the Affymetrix probe ID. Columns 2 to 4 (labeled as “WT1”, “WT2” and “WT3”) are the measurements of fluorescence intensity in the three different biological replicates of the wild-type group. Columns 5 to 7 (labeled as “P-/- 1”, “P-/- 2” and “P-/- 3”) are the measurements of fluorescence intensity in the three different biological replicates of the *parp-1*^-/-^ group. All measurements are presented as a log_2_ value. Column 8 corresponds to the fold difference between both groups, i.e., negative values for genes downregulated in *parp-1^-/-^* and positive values for genes upregulated in *parp-1^-/-^*.

***Supplementary Table 2***

List of 602 genes significantly misregulated in the *parp-1^-/-^* group compared to wild type. The first column corresponds to the Flybase ID, and second column is the *Drosophila melanogaster* gene symbol. Columns 3 to 5 (labeled as “WT1”, “WT2” and “WT3”) are the measurements of fluorescence intensity in the three different biological replicates of the wild-type group. Columns 6 to 8 (labeled as “P-/- 1”, “P-/- 2” and “P-/- 3”) are the measurements of fluorescence intensity in the three different biological replicates of the *parp-1*^-/-^ group. All the measurements are presented as a log_2_ value. Column 9 corresponds to the fold difference between both groups, i.e., negative values for genes downregulated in *parp-1^-/-^* and positive value for genes upregulated in *parp-1^-/-^*.

***Supplementary Table 3.***

Comparison of fold differences obtained from microarray and quantitative RT-PCR. Column 1 presents the 10 DEGs selected to be tested by quantitative rt-PCR. Column 2 presents the fold difference obtained with microarray. Column 3 presents the fold difference obtained by quantitative rt-PCR. A two tailed *t*-test based on the three biological replicates was performed. ***: p-value < 0.01, *: p-value < 0.05. The differences in range between microarray and qRT-PCR may have resulted from the difference in techniques. However, all tested genes significantly downregulated in the *parp-1^-/-^* group in microarray were also significantly downregulated in qRT-PCR. Conversely, the genes upregulated in microarray were all significantly upregulated in qRT-PCR. Taken together, these results suggest that the results obtained from microarray are reproducible.

***Supplementary Table 4***

List of DEGs with unknown function. The first column corresponds to the Flybase ID, and the second column is the *Drosophila melanogaster* gene symbol. Column 3 is the fold difference between wild-type and *parp-1^-/-^* groups, i.e., negative values for genes downregulated in *parp-1^-/-^* and positive value for genes upregulated in *parp-1^-/-^*.


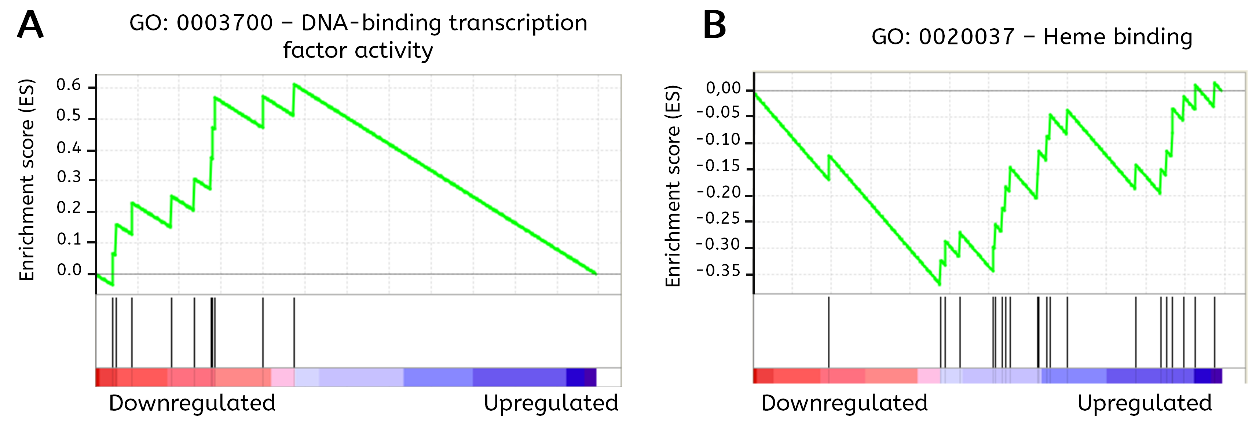


***Supplementary Figure 1***

Examples of results obtained with Gene Set Enrichment Analysis (GSEA) software. **A**) Calculation of the score for the Gene ontology (GO)-term ‘GO: 0003700 – DNA-binding transcription factor activity’. **B**) Calculation of the score for the GO-term ‘GO:0020037 – Heme binding’. First, GSEA sorts all differentially expressed genes (DEGs) according their differential expression between the wild type and *parp-1* knockout groups from the most downregulated to the most upregulated in the *parp-1* knockout group (color shade from red (downregulated) to blue (upregulated)). The list created is called *L*. Then, GSEA calculates a score (called enrichment score (ES)) for each GO-term. The score is calculated by walking down *L*, increasing a running-sum statistic when GSEA encounters genes associated with this GO-term and decreasing it when GSEA encounters genes not associated with this GO-term (green curves). The magnitude of the increment depends on the position of the genes in *L*. That is, the increment is higher if the gene is at the extreme *L*, rather than closer to the median. The score is the maximum deviation from zero encountered in the random walk, and it corresponds to a weighted Kolmogorov-Smirnov-like statistic [**71**, **72**] (herein, the ES score is 0.61 for the GO-term ‘DNA-binding transcription factor activity’ (**A**) and - 0.37 for the GO term ‘Heme binding’ (**B**)). After that, GSEA performs a set of random permutations between groups, generating a null distribution. The p-value of the score of each GO-term is then calculated relative to this null distribution. Finally, GSEA calculates the FDR for each GO-term based on their score and the number of associated DEGs.
