# Supplementary material for: Poly(ADP-ribose) polymerase 1 in genome-wide expression control in Drosophila
Source: Sci Rep. 2020 Dec 3;10:21151. doi: 10.1038/s41598-020-78116-5 (PMC7712786; doi:10.1038/s41598-020-78116-5)
Supplement: Supplementary file 4 — Supplementary Table 3. [file 41598_2020_78116_MOESM4_ESM.docx]

| Gene | Family/Function | Fold difference (microarray) | Fold difference (qRT-PCR) |
| --- | --- | --- | --- |
| CG3588 | Unknown | 836.10 | 188.30**^***^** |
| Cyp6w1 | Cytochrome P450 | 434.77 | 98.84**^***^** |
| ninaD | Rhodopsin biosynthesis | 1533.24 | 87.34**^***^** |
| alpha-Est7 | Insecticide tolerance | 88.57 | 22.50**^***^** |
| CG11893 | Unknown | -282.53 | -18.33**^*^** |
| MtnC | Heavy metal detoxification | -50.12 | -16.68**^***^** |
| Cyc | bHLH-PAS transcription factor | -3.69 | -2.48**^***^** |
| NFAT | Nuclear factor activated T-cells transcription factor | -5.52 | -1.49**^***^** |
| ac | bHLH transcription factor | -2.92 | -1.60**^***^** |
| Eip78C | Nuclear receptor transcription factor | -63.68 | -5.24**^*^** |
